# Evaluation of the *in vitro* and *in vivo* antioxidant potential of *Punica granatum* L. against toluene-induced liver injuries in rats

**DOI:** 10.14202/vetworld.2022.374-382

**Published:** 2022-02-17

**Authors:** Fatma Zohra Arkoub, Leila Hamdi, Labiba Kahalerras, Meriem Hamoudi, Kamel Khelili

**Affiliations:** 1 Department of Biology, Laboratory of Animal Ecophysiology, Faculty of Sciences, University Badji Mokhtar-Annaba, Annaba 23000, Algeria; 2 Department of Biology, Laboratory of Phytotherapy Applied to Chronic Diseases, Faculty of Natural and Life Sciences, University Ferhat Abbas Setif 1, Setif 19000, Algeria

**Keywords:** antioxidant activity, liver injuries, oxidative stress, *Punica granatum*, toluene

## Abstract

**Background and Aim:**

Humans are more frequently exposed to chemicals in daily life by inhalation of indoor and outdoor air. However, abusers and workers are the most exposed to those chemicals and their health risks particularly, liver diseases. The present study investigated the protective effects of pomegranate juice (PJ) (*Punica granatum*) and pomegranate peel aqueous extract (PAE) supplementation against toluene (Tol)-induced hepatotoxicity in *Wistar* rats.

**Materials and Methods:**

A phytochemical analysis and assessment of the 2,2-diphenyl-1-picrylhydrazyl (DPPH) scavenging activity were performed for the PJ and the PAE. The *in* *vivo* study was carried out using 70 *Wistar* rats that were divided into seven groups, each consisting of 10 rats. All groups were treated orally for 6 weeks as follows: Control (C), positive controls (CO: 1.25 mL/kg body weight [BW]; PJ: 4 mL/kg BW; and PAE: 400 mg/kg BW), Tol (550 mg/kg BW), and a mixture each of PJ-Tol and PAE-Tol. At the end of the 45^th^ day of study, the hepatic biochemical markers (transaminases, alkaline phosphatase, total bilirubin, albumin, and total proteins) were auto-analyzed, as well as histology and oxidative stress markers of the liver were evaluated.

**Results:**

The phytochemical analysis revealed that the DPPH scavenging activity and the total phenolic, flavonoid, and tannin contents were higher in the pomegranate peel extract versus the juice. The results also showed that Tol significantly increased liver enzyme activities and total bilirubin levels, whereas albumin and total proteins were significantly decreased. Similarly, Tol provoked a significant increase in hepatic malondialdehyde levels, with a decrease in glutathione content and glutathione peroxidase activity. The biochemical changes agreed with the hepatic histological alterations. A significant improvement in all parameters was observed in the PAE-Tol group compared with the PJ-Tol group.

**Conclusion:**

Exposure to Tol altered the hepatic antioxidant and biochemical parameters and histological profile of the rats, and PAE was more powerful than PJ in reducing Tol liver injuries through its antioxidant activity.

## Introduction

At present, the return to natural products has become one of the significant directions of several research projects. Plant substances and extracts, as well as their essential oils, seeds, and peel powders, have been demonstrated to play basic roles in the prevention or attenuation of the physiological disruptions stemming from exposure to different chemicals [1]. Among the plants that have attracted great interest, *Punica granatum*, which is popularly known as pomegranate, is a small fruit tree of the Punicaceae family that grows in countries bordering the Mediterranean Sea, Pakistan, India, and Iran [2]. Different parts of the pomegranate plant, and especially its fruits, have been widely used in traditional medicine as a remedy for several diseases and symptoms. It is claimed to be a health-promoting food because of the great number of potentially active nutrients, which are found mainly in its juice and peel [3]. The main bioactive constituents of pomegranate are polyphenols, flavonoids, and tannins, which contribute to its antioxidant activity and render it a potential source of natural antioxidants. In fact, the antioxidant activities of pomegranate seem to be 3 times higher than those of green tea [4]. The fruits of *P. granatum* are also reported to possess several broad-spectrum biological functions, such as neuroprotective, cardioprotective, immune-modulatory, anticarcinogenic, antibacterial, antiapoptotic, and anti-inflammatory activities [4,5] Several lines of evidence have implicated oxidative stress and inflammation in the development of many deleterious effects, particularly liver disease. Among the hepatic pathologies, toxicity is the most common risk factor, which is usually attributed to the use of drugs, alcohol, and other chemical agents [6,7]. Toluene (Tol) is the most consumed volatile substance with a high potential for different types of abuse [8]. It is most often inhaled nasally (sniffing), but it can also be taken orally (huffing) [9]. After absorption, the lipophilic nature of Tol enables it to easily pass and spread through the body, mainly in highly vascularized and lipid-rich tissues [10]. High concentrations of Tol were detected in the brain and liver tissues of individuals who died from glue-sniffing [11]. Moreover, it has been demonstrated that the main effect of Tol is to disrupt the function of the nervous system. However, it triggers many other damaging outcomes, such as embryo- and genotoxicity, teratogenicity, neurobehavioral changes, and renal and hepatic failure in experimental organisms [10,12,13].

*In vitro* and *in vivo* experiments have shown that Tol severely damages DNA and causes an imbalance in the pro-oxidant/antioxidant status [13,14]. Once absorbed, Tol is hydroxylated in the liver by the isoenzymes of cytochrome P450, principally by CYP 2EI and weakly by CYP 286, CYP2C8, and CYP 1A2, yielding hippuric acid, which is the most important metabolite of Tol [15]. According to the previous study, the metabolism of Tol is believed to be the major cause of the large increase in reactive oxygen species (ROS) through the formation of Tol epoxides with high reactivity, resulting in inflammation, genetic material damage, and subsequently leading to cell death [16].

Based on the facts stated above, the study aimed to shed additional light on the possible changes that occur in hepatic histological, biochemical, and antioxidant profiles after subchronic exposure to Tol. We also demonstrated the protective potential of *P. granatum* juice and peel aqueous extract (PAE) against solvent-induced hepatotoxicity in male *Wistar* rats.

## Materials and Methods

### Ethical approval

Experimental procedures were performed according to the National Guide for the Care and Use of Laboratory Animals and approved by the Animal Sciences Ethical Committee of Badji Mokhtar Annaba University-Algeria.

### Study period and location

This study was conducted from November 2018 to February 2019. At Animal House, Department of Biology, Faculty of Sciences, University of Badji Mokhtar Annaba-Algeria.

### Chemicals

Tol 99.5% and all other chemical products used in this study were purchased from Sigma-Aldrich, USA).

### Fruit collection

Mature *P. granatum*, common fruits were collected in November 2018 from a local area (Besbes-El-Taref) with the following geographic coordinates: Latitude, 36.671028 and longitude, 7.773417; 36° 40’ 15.7” North, 7° 46’ 24.3” East. The whole fruit was washed and manually peeled without separating the seeds. Pomegranate juice (PJ) was prepared daily using a commercial centrifugal blender (Sinbo, Turkey). The peels were kept, dried in the shade, and finally ground to a powder. The powder was soaked in distilled water for 3 days at room temperature (22±2°C) with occasional shaking, after which the extract was filtered through cotton gauze.

### Antioxidant activity

#### 2,2-Diphenyl-1-picrylhydrazyl (DPPH) radical scavenging assay

The free radical scavenging activity of the extracts was measured using the DPPH assay [17]. After dissolving the extracts in methanol, a solution of DPPH in methanol (0.04 mg/mL) was prepared, and 1250 μL of this solution was added to 50 μL of the fraction solution at different concentrations. The mixture was shaken vigorously and then kept in the dark for 30 min at 22±2°C followed by the measurement of the absorbance at 517 nm using BHT as a standard. Radical scavenging activity was calculated using the following equation:

Radical scavenging activity (%) = (A blank–A sample/A blank) ×100

### Determination of the total polyphenol content

The total polyphenol content was determined using the Folin–Ciocalteu method, as described by Li *et al*. [18]. Briefly, 0.1 mL of extract was mixed with 0.5 mL of Folin–Ciocalteu reagent (diluted 10-fold). After a 4 min incubation, 0.4 mL of a 7.5% sodium carbonate (Na_2_CO_3_) solution was added. The final mixture was shaken and then incubated for 1 h in the dark at 22±2°C. The absorbance of the samples was measured at 760 nm and the results are expressed as microgram of gallic acid equivalents (GAEs) per milligram of dried extract (mg GAE/g extract).

### Determination of total flavonoid content

The total flavonoid content of each extract was determined using a colorimetric method as described by Mouffouk *et al*. [19]. Briefly, 1 mL of extract was mixed with 1 mL of an aluminum chloride solution (2%) and allowed to stand for 10 min. The absorbance of the mixture was then determined at 430 nm versus a blank equivalent to the prepared extract. The results are expressed as micrograms of quercetin equivalent per milligram of dried extract (mg QE/g extract).

### Determination of total tannin content

Total tannins were measured using the method of Folin-Denis [20]. Briefly, 1 mL of extract and a standard solution of tannic acid (100-800 μg/mL) were brought to 7.5 mL with distilled water. Subsequently, 0.5 mL of Folin-Denis reagentand 1 mL of Na_2_CO_3_ solution were added. The volume was made up to 10 mL with distilled water, and the absorbance was read at 700 nm. The total tannin content was expressed as mg of tannic acid equivalent (mg TAE)/100 g of extract.

### *In vivo* experimental design

Seventy adult male *Wistar* rats (230±30 g) were obtained from the Pasteur Institute (Algiers). The rats were placed in the animal housing facility of the biology department, where they were allowed to acclimate for 3 weeks under standard laboratory conditions. The animals were fed commercial pellets and given tap water *ad libitum*.

The first group served as the control and received distilled water only (C); the second, third, and fourth groups served as positive controls and received 1.25 mL/kg body weight (BW) of corn oil (CO), 4 mL/kg BW of PJ, and 400 mg/kg BW of pomegranate PAE, respectively; the fifth group (Tol) received 550 mg/kg BW of Tol diluted in corn oil, as a vehicle; the sixth group was treated with Tol (550 mg/kg BW), followed by PJ 2 h later; finally, the seventh group was treated with an equal dose of Tol, followed by PAE 2 h later. All groups were treated daily by gavage for six weeks.

### Sample collection

On 45^th^ day the rats were sacrificed by decapitation and blood samples (2 mL) were immediately collected in dry tubes. Serum was obtained by centrifugation at 3000× *g* for 10 min and was then stored at –20°C until biochemical analysis. The liver was dissected and cleaned from adipose tissues, and a fragment was stored at –20°C for the assessment of oxidative stress parameters. Another fragment was fixed in a 10% formalin solution to prepare histological sections.

### Determination of biochemical parameters

The activities of serum alanine aminotransaminase (ALT), aspartate aminotransaminase (AST), and alkaline phosphatase (ALP), and the levels of serum albumin, total proteins, and total bilirubin were measured in the Clinical Chemistry Laboratory using an automated random access clinical chemistry analyzer (ERBA XL-600, ERBA Diagnostics Mannheim GmbH, Mannheim, Germany).

### Determination of oxidative stress parameters

Glutathione (GSH) levels in a liver homogenate were assessed spectrophotometrically according to the method of Weckbecker and Cory [21], by monitoring yellow color development against the blank reagent. Glutathione peroxidase (GPx) activity was determined using the method of Flohé and Günzler [22]. This assay is based on the reduction of hydrogen peroxide (H_2_O_2_) in the presence of reduced GSH. The level of lipid peroxidation was measured through malondialdehyde (MDA) using the method of Ohkawa *et al*. [23], which is based on the determination of the thiobarbituric acid reaction.

### Determination of tissue proteins

Protein concentration was determined according to the method of Bradford [24] using bovine serum albumin as a standard.

### Histopathological study

Formaldehyde-fixed samples were dehydrated in a graduated series of alcohols at increasing degrees, from 70° to 100°, using a circulating inclusion automaton (Thermo Scientific, Microm STP 120, UK) and incorporated in kerosene (paraffin). Paraffin sections (5 µm thick) were mounted on a clean glass slide and stained with hematoxylin and eosin (H&E) (BioLab, Mumbai, India) for light microscopic examination (×40) (Leica DMLB, Wetzlar, Germany) [25].

### Statistical analysis

Statistical significance was determined using a one-way analysis of variance with Tukey’s *post hoc* test. Data are reported as the mean±standard error of the mean using GraphPad Prism 7 (GraphPad Software, San Diego, CA, USA) and Statistical Package for the Social Sciences SPSS version 26.0 for Windows (IBM Corporation, Armonk, NY, USA), with significance set at p<0.05.

## Results

### DPPH scavenging activity

**Figure-1:**
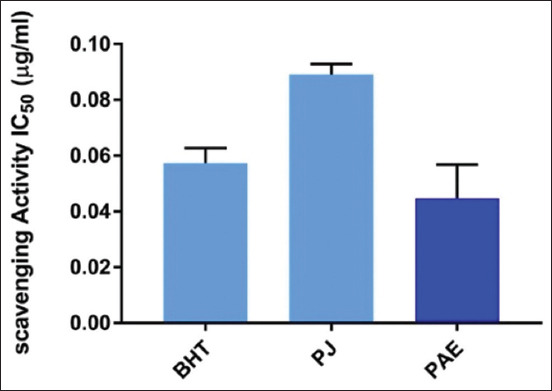
DPPH scavenging activity of Punica granatum juice and peel aqueous extract (mean±standard error of the mean). DPPH=2,2-Diphenyl-1-picrylhydrazyl, BHT=Butylated hydroxytoluene, IC_50_=Inhibitory concentration, PJ=Pomegranate juice, PAE=Peel aqueous extract.

The assessment of the antioxidant activity of *P. granatum* juice and PAE is reported in Figure-1. The DPPH scavenging activity assay demonstrated that the PAE had higher activity than the PJ.

### Total phenolic, flavonoid, and tannin contents

The total phenolic, flavonoid, and tannin contents in PAE and PJ are reported in Table-1. According to these results, the amount of total phenolic compounds, flavonoid, and tannin was much greater in the PAE (0.78±0.01 mg GAE/g, 0.49±0.002 mg EQ/g, and 0.74±0.033 mg TAE/100 g, respectively) compared with the PJ (0.52±0.005 mg GAE/g, 0.22±0.029 mg EQ/g of flavonoids, and 0.44±0.042 mg TAE/100 g).

**Table 1:** Total phenolic, flavonoids, and tannins content of *Punica granatum* juice and peel aqueous extract (mean±standard error of the mean).

	Total phenolic (mg GAE/g)	Flavonoids (mg EQ/g)	Tannins (mg TAE/100 g)
PJ	0.52±0.005	0.22±0.029	0.44±0.042
PAE	0.78±0.01	0.49±0.002	0.74±0.033

GAE=Gallic acid equivalent, EQ=Quercetin equivalent, TAE=Tannic acid equivalent, PJ=Pomegranate juice, PAE=Peel aqueous extract

### Liver weight

**Figure-2:**
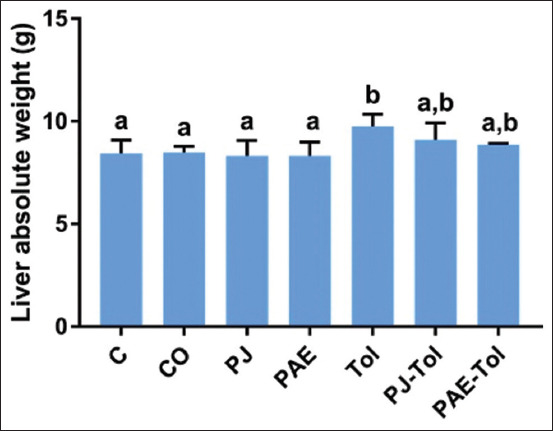
Liver absolute weight of rats in different experimental groups (mean±standard error of the mean). Values that do not share the same letters differ significantly at p<0.05. C=Control, CO=Corn oil, PJ=Pomegranate juice, PAE=Peel aqueous extract, Tol=Toluene, PJ-Tol=Toluene+pomegranate juice, PAE-Tol=Toluene+peel aqueous extract.

The data analysis indicates a significant increase in the liver absolute weight in the Tol-treated group compared with the control and positive control groups. In contrast, insignificant changes were observed in the liver weight of the PJ-Tol and PAE-Tol-treated groups compared with the Tol group (Figure-2).

### Serum biochemical parameters

The biochemical parameters of the liver are summarized in Table-2. Compared with the controls, a statistically significant increase in the serum AST, ALT, ALP, and total bilirubin levels was recorded in the Tol group. Interestingly, *P. granatum* supplementation significantly improved the activity of enzymes in the PAE-Tol group compared with the Tol group.

**Table 2:** Plasma biochemical parameters of rats in different experimental groups (mean±standard error of the mean).

Groups	AST (µL/L)	ALT (µL/L)	ALP (µL/L)	Albumin (g/L)	Total protein (g/L)	Total bilirubin (mg/L)
C	138.60±3.91^a^	31.95±0.91^a^	86±6.17^a^	33.82±1.18^a^	92.63±2.49^a^	5.76±0.09^a^
CO	139.80±2.25^a^	31.43±0.97^a^	86.89±2.66^a^	33.79±1.55^a^	91.07±5.50^a^	5.65±0.13^a^
PJ	138.60±3.48^a^	31.65±0.70^a^	86.83±2.17^a^	34,17±0.55^a^	93.11±1.47^a^	5.59±0.46^a^
PAE	138.30±0.54^a^	31.50±1.25^a^	86.24±6.89^a^	34.38±1.01^a^	93.38±2.88^a^	5.75±0.39^a^
Tol	175.70±4.09^c^	47.63±0.64^c^	129.20±7.95^c^	26.48±1.05^c^	71.65±2.40^b^	8.30±0.97^b^
PJ-Tol	161±6.65^bc^	40.33±0.88^b^	106±4.54^ab^	29.46±1.06^ab^	81.01±4.95^ab^	7.30±0.52^ab^
PAE-Tol	157.50±1.18^b^	37.71±0.30^b^	96.25±2.56^a^	31.94±1.54^ab^	87.54±0.52^a^	6.53±0.32^ab^

Values that do not share the same letters differ significantly at *P<*0.05. C=Control, CO=Corn oil, PJ=Pomegranate juice, PAE=Peel aqueous extract, Tol=Toluene; PJ-Tol=Toluene+pomegranate juice, PAE-Tol=Toluene+peel aqueous extract, AST=Aspartate aminotransaminase, ALT=Alanine aminotransaminase, ALP=Alkaline phosphatase

Conversely, treatment with Tol produced a significant decrease in the serum levels of albumin and total proteins compared with the control and positive control groups. Nevertheless, this decrease almost reached the normal level after PAE administration compared with the rats treated with Tol. PJ supplementation increased the activity of liver enzymes slightly.

### GSH and GPx activity

**Figure-3:**
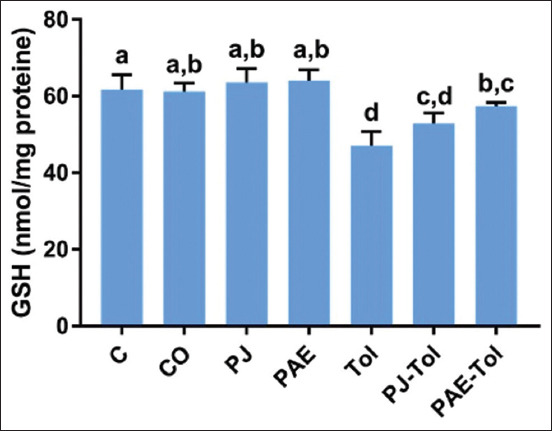
Variation of hepatic GSH levels (nmol/mg proteins) in different experimental groups (mean±standard error of the mean). Values that do not share the same letters differ significantly at p<0.05. C=Control, CO=Corn oil, PJ=Pomegranate Juice, PAE=Peel aqueous extract, Tol=Toluene, PJ-Tol=Toluene+pomegranate juice, PAE-Tol=Toluene+peel aqueous extract.

**Figure-4:**
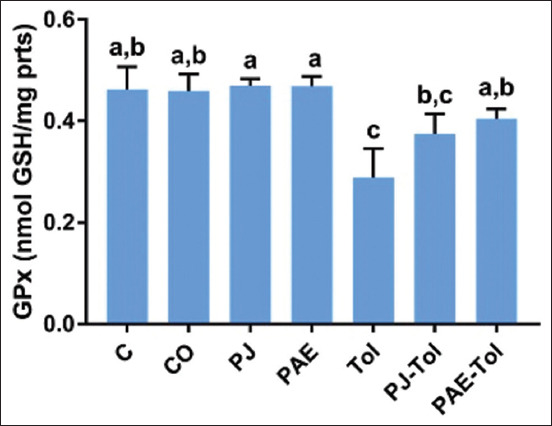
Variation of hepatic GPx activity (nmol GSH/mg protein) in different experimental groups (mean±standard error of the mean). Values that do not share the same letters differ significantly at p<0.05. C=Control, CO=Corn oil, PJ=Pomegranate juice, PAE=Peel aqueous extract, Tol=Toluene; PJ-Tol=Toluene+pomegranate juice, PAE-Tol=Toluene+peel aqueous extract.

As shown in Figures-3 and 4, exposure of rats to Tol significantly depleted the hepatic GSH pool and GPx activity compared with the control and positive control groups. However, the administration of *P. granatum* PAE restored the antioxidant profile compared with the Tol exposed group. The groups supplemented with PJ did not show significant differences compared with the Tol group.

### Lipid peroxidation

**Figure-5:**
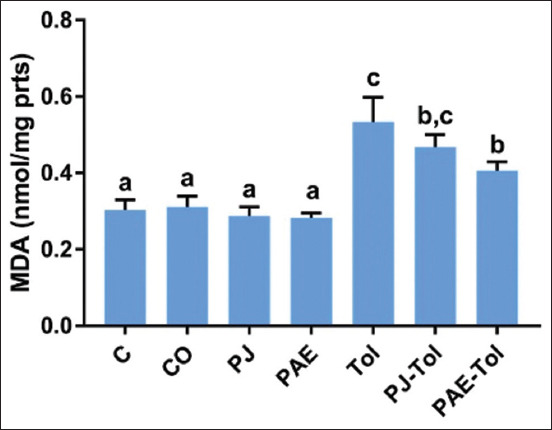
Variation of hepatic MDA concentration (nmol/mg proteins) in different experimental groups (mean±standard error of the mean). Values that do not share the same letters differ significantly at p<0.05. C=Control, CO=Corn oil, PJ=Pomegranate juice, PAE=Peel aqueous extract, Tol=Toluene, PJ-Tol=Toluene+pomegranate juice, PAE-Tol=Toluene+peel aqueous extract.

Figure-5 shows that the liver MDA content was significantly increased in the Tol-treated group compared with the control and positive control groups. The MDA levels of PAE supplemented rats were lower than those the groups treated with Tol. There was a slight decrease in MDA levels in the PJ-Tol group compared with the Tol group.

### Gross examination

Grossly, the liver of the animals in the control and positive control groups (CO, PJ, and PAE) exhibited smooth surfaces and did not show any sign of nodules. In contrast, the liver of the animals in the Tol group lost its normal architecture, as assessed by the presence of regenerating nodules and cysts on its surface, with thickening and hardening of the liver borders. No abnormal appearance or macroscopic nodules were visible on the surface of the liver of animals in the PJ-Tol and PAE-Tol groups (Table-3).

**Table 3:** Gross liver of different experimental groups, control, and positive control rats (CO, PJ, and PAE) showing normal liver, Tol-induced rats showing rough, nodular, and irregular appearance of liver external surface, PJ-Tol and PAE-Tol rats illustrating a nearly smooth surface.

Experimental groups						

C	CO	PJ	PAE	Tol	PJ-Tol	PAE-Tol
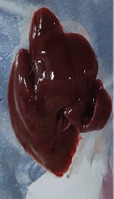	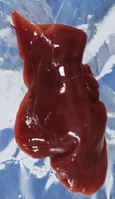	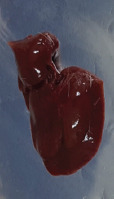	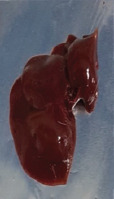	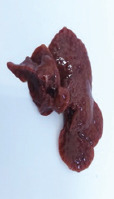	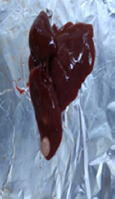	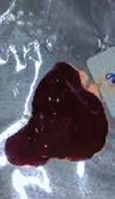

C=Control, CO=Corn oil, PJ=Pomegranate juice, PAE=Peel aqueous extract, Tol=Toluene, PJ-Tol=Toluene+pomegranate juice, PAE-Tol=Toluene+peel aqueous extract

### Histological examination

**Figure-6:**
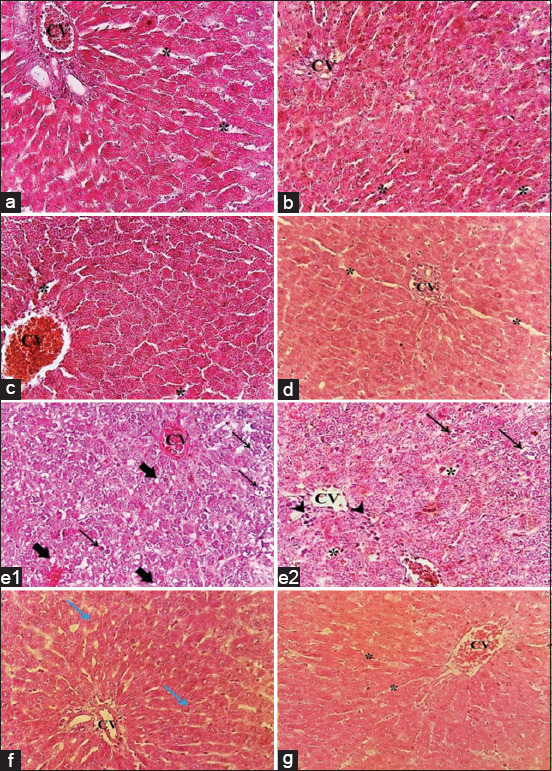
Histopathology light microphotographs of liver from different experimental groups. Control (a), CO (b), PJ (c), and PAE (d) showing the normal histological structure of the central vein and surrounded by blood sinusoids (*) (×40). Tol group showing (e1) ballooning degeneration of hepatocytes (black arrow) with steatosis (thick arrow), (e2) inflammatory cells infiltration (arrowhead) in the portal area (×40). PJ-Tol (f) and PAE-Tol (g) groups showing a few degenerative changes and diffuse Kupffer cells proliferation (blue arrow) in between the hepatocytes (×40). C=Control, CO=Corn oil, PJ: Pomegranate juice, PAE=Peel aqueous extract, Tol=Toluene, PJ-Tol=Toluene + Pomegranate juice, PAE-Tol=Toluene + Peel aqueous extract.

As shown in Figure-6, liver sections stained with H&E revealed a normal histological structure of the central vein and surrounding hepatocytes in the control group (A). Moreover, the sections of the liver from rats treated with CO (B), PJ (C), and PAE (D) were similar to the control. In the Tol-treated group, infiltration of inflammatory cells, ballooning degeneration of hepatocytes, and steatosis were recorded ((E1)–(E2)). In contrast, the PJ-Tol and PAE-Tol groups showed mild-to-moderate injury.

## Discussion

The liver is a remarkable organ that acts as the chief player in the metabolism and biotransformation of many toxic compounds through various chemical reactions. Thus, it targets many types of impairment that produce hepatotoxicity and cause health complications [26].

Exposure to Tol at 550 mg/kg BW daily for 45 days yielded a significant increase in the liver absolute weight. This could be attributed to an increase in the activity of inflammatory agents that could have resulted in the inflammation of liver tissues. It has recently been shown that exposure to gasoline containing Tol provoked an elevation of the levels of pro-inflammatory cytokines, such as interleukin (IL)-1β, IL-6, tumor necrosis factor-α, and interferon-γ [27].

Serum AST, ALT, and ALP have a predominantly hepatic origin in the body, they are often considered to be the most sensitive indicators of liver injury. The activities of hepatic enzymes recorded in this study were much higher in the Tol-treated group. Such results are comparable to previously reported work [28], which documented a clear increase in hepatic enzymes in response to Tol treatment. This increase might be attributed to an elevation in the oxidative metabolism of the liver, resulting in membrane degeneration, as well as cholestatic damage, leading to the leakage of enzymes into the blood serum [29]. However, the elevated bilirubin levels observed could indicate decreased hepatic conjugation or overproduction of bilirubin caused by hemolysis [30-32].

Consistently, Tol causes a decline in albumin and total protein levels, which might be linked to the obstruction of protein biosynthesis and free amino acid metabolism in the liver [33]. It was established that exposure to Tol could promote cell apoptosis by elevating pro-apoptotic protein (Bax) levels and caspase-3 activity in the liver [14,34]. The AMP-activated protein kinase has also been associated with the mechanism involved in cell apoptosis in response to ROS, such as H_2_O_2_ [35].

Several lines of evidence obtained from the current experiment suggest that ROS causes the toxic effect of Tol on the liver. The ratio of liver GSH and GPx was markedly decreased in rats exposed to Tol, which was accompanied by an increase in MDA levels. These results agreed with the literature reporting that inhalation of Tol raised hepatic MDA concentration and diminished GPx activity [36]. Furthermore, it was previously found [14] that the exposure of rats to Tol reduced the activity of hepatic antioxidant enzymes. It is known that the Tol metabolism is a major intracellular ROS generator in the liver tissues [16], where it occurs through nicotinamide adenine dinucleotide phosphate oxidation by the mitochondria electron transport chain [29]. The results obtained here were possibly linked to the Tol-induced increase in ROS generation, leading to lipid peroxidation and antioxidant depletion, which, in turn, contributes to the occurrence of hepatic apoptosis [37]. It can be speculated that the marked decreases in GSH concentration were related not only to the overproduction of ROS but could also result from impaired regeneration by GSH reductase. Considering the fact that mitochondria occupy 18% of hepatocytes [38], they are targets of drug toxicity, causing mainly oxidative stress. In response to this stress, the permeability of the mitochondrial membrane may increase, which may induce mitochondrial dysfunction and declined metabolic regeneration of reducing equivalents, resulting in decreased regeneration of GSH [39]. In the same context, many investigators have postulated that Tol produces high levels of H_2_O_2_ and NO, which override enzymatic activity, thus inducing lipid peroxidation, DNA oxidation, and oxidation or nitrosylation of thiols [14,40].

The disruptions of liver function through oxidative stress injury outlined here were affirmed by the histological outcomes of rats treated with Tol. The main histopathological features of these animals included infiltration of leukocytes, steatosis, degenerative changes, and other manifestations. Tol was also reported to cause slight pericentral fibrosis and ballooning degeneration [28], as well as sinusoid dilation, hemorrhage, inflammatory cell infiltration, vacuolization, and necrosis [41]. Moreover, hepatocyte degeneration and apoptosis, as well as inflammatory cell infiltration, were reported in the Tol-induced toxicity of rats, which could be explained primarily by the formation of ROS [14].

Notably, *P. granatum* supplementation generally restored the deterioration of liver tissues triggered by Tol. Pomegranate has been suggested to play a mitigating role against liver damage because of its high content of phenolic compounds, phenolic acids, ellagic tannins, flavonoids, and anthocyanins (delphinidin, cyaniding, and pelargonidin), which possess many prominent capacities [42,43]. The i*n vitro* and *in vivo* antioxidant tests performed here proved that PAE displayed the highest amount of total phenolic compounds, flavonoids, and tannins, with elevated free radical scavenging activity. These results are in harmony with the previous reports indicating that pomegranate peels and pulps have a higher total phenolic compound content and antioxidant activity than PJ [44,45]. It is well documented that hydrolyzable tannins are the most prominent constituents in the context of pomegranate antioxidant capacity.

As observed in the present study, *P. granatum* has a considerable free radical scavenging ability; thus, it seems that the protective potential of pomegranates may depend on the alleviation of oxidative stress damage. This effect has been affirmed by the modulation of serum liver biomarkers and structural organization, decreased MDA levels, and enhanced GSH and GPx activity, especially in rats that received PAE, which agrees with the previous results [46,47]. The high potential of phenolic components to scavenge radicals might be explained by their ability to donate a hydrogen atom from their phenolic hydroxyl groups [48]. It has been demonstrated that the active constituents of pomegranate, such as flavonoids and ellagic acid, restored the oxidant/antioxidant balance by increasing C-glutamyl cysteine synthetase, which is the critical enzyme in GSH synthesis [49]. The authors also found evidence that punicalagin extracted from PJ and peel increases serum paraoxonase activity, which may protect against lipid peroxidation [50]. Moreover, antioxidant activity might not be the only factor responsible for the ability of pomegranates to reduce the severity of Tol hepatotoxicity; another potential therapeutic tool is linked to its anti-inflammatory and anti-apoptotic activity. It was revealed that pomegranate peel extract can reduce hepato-inflammation and oxidative stress by suppressing the production of pro-inflammatory cytokines and enhancing Nrf2 activation [47]. Thus, Nrf2 upregulation leads to an elevation in the expression of antioxidant enzymes, to combat ROS generation [51]. This action could be attributed to bioactive elements, such as punicalagin and punicalin, which contribute to nuclear factor kappa B (NF-κB) downregulation by blocking NF-κB-driven transcription, in turn, diminishing inducible nitric oxide synthase and subsequent nitric oxide production [52]. Furthermore, pomegranates are also able to downregulate caspase-3 and upregulate the antiapoptotic protein Bcl-2; therefore protecting cells from death [48].

## Conclusion

The *in vivo* experiments confirmed that exposure to a moderate dose of Tol elicits liver injury, as established based on histological alterations, lipid peroxidation, and serum biochemical disruptions. However, supplementation with *P. granatum* juice and peel extract almost normalized most of the markers. The *in vitro* tests demonstrated that PJ and PAE possess important components such as polyphenols, flavonoids, and tannins with antioxidant potential. Thus, it can be concluded that exposure to Tol may cause hepatic alterations; whereas the consumption of pomegranate seems to partially (Pomegranate juice did not mitigate the hepatotoxicity as the peel extract did) attenuate the toxic effects of this solvent and these beneficial effects are possibly due to its content in phenolic compounds. However, further research is needed to ascertain the precise mechanisms of action of Tol on mitochondrial function, as well as to give an appropriate dose recommendation of *P. granatum* supplementation for humans.

## Authors’ Contributions

FZA: Designed and performed the experiments, interpreted and analyzed the data, and wrote the manuscript. LH: Corrected and revised the manuscript. LK: Participated in the experiments and the analysis of results. MH: Supervised the experiments that were performed in their respective laboratory. KK: Supervised the work and revised the manuscript. All authors read and approved the final manuscript.
